# Osteomyelitis of the maxilla caused by *Actinomyces* sp.

**DOI:** 10.1590/0100-3984.2017.0062

**Published:** 2018

**Authors:** Lívia de Oliveira Antunes, Rafael da Silveira Borges, Wania Vasconcelos de Freitas, Simone Rachid de Souza, Diogo Goulart Corrêa

**Affiliations:** 1 Hospital Casa de Portugal, Rio de Janeiro, RJ, Brazil.; 2 Hospital Federal do Andaraí, Rio de Janeiro, RJ, Brazil.; 3 Hospital Casa de Portugal e Universidade Federal do Rio de Janeiro (UFRJ), Rio de Janeiro, RJ, Brazil.

*Dear Editor,*

**Figure 1:**
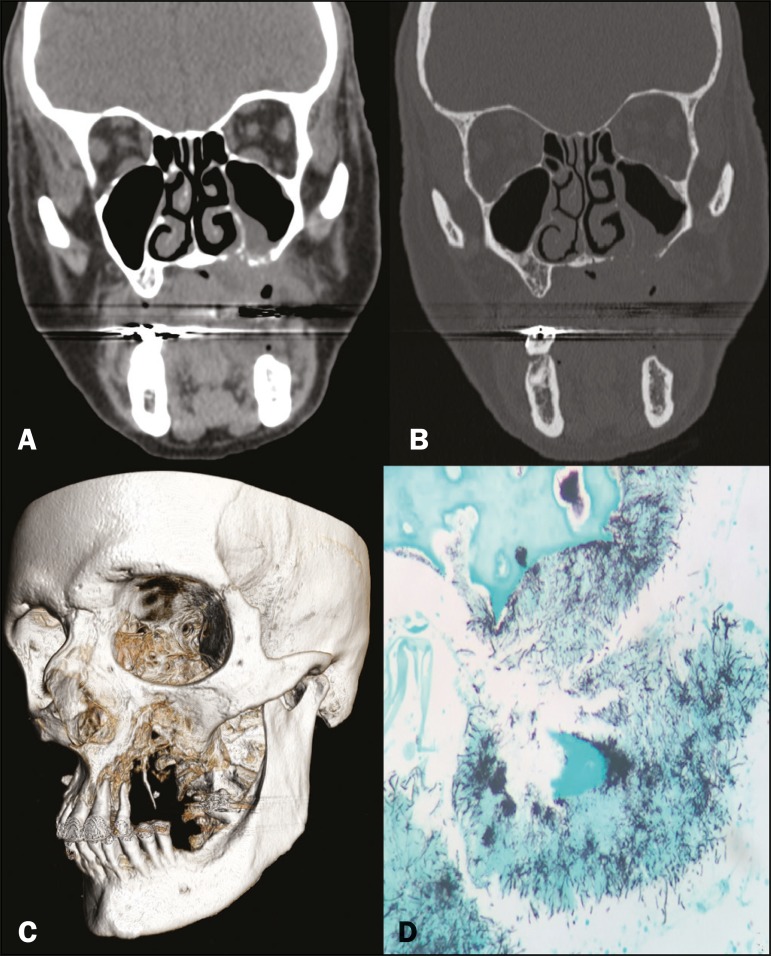
Computed tomography of the facial sinuses, with a soft-tissue window (A) and a bone window (B), reconstructed in the coronal plane, showing a soft-tissue density lesion eroding the maxilla, the floor of the maxillary sinus, and the left side of the palate, as well as forming a fistula from the oral cavity to the nasal cavity and to the left maxillary sinus. Three-dimensional reconstruction of a computed tomography scan (C), showing bone erosion in the maxilla and left palate. Grocott’s staining (D) showing colonies of filamentous *Actinomyces* bacteria interspersed with bone tissue (magnification, ×400).

We report the case of a 76-year-old female patient with diabetes and hypertension that were not being treated on a regular basis. She had undergone a tooth extraction, then continued to feel pain and had a persistent low fever, even during the course of oral antibiotic therapy. Over the following months, she lost multiple, contiguous, teeth at the previously manipulated site. Computed tomography for investigation of bone involvement showed soft-tissue density that was poorly defined, indicating bone erosion in the left maxilla, extending to the maxillary sinus, and palatal fistula. A biopsy of the lesion showed mixed inflammatory infiltrate with granulation tissue (visualized with hematoxylin-eosin staining) and actinomycete colonies permeating the bone tissue (visualized with Grocott’s staining), which allowed us to make a diagnosis of osteomyelitis caused by *Actinomyces* sp. (Figure 1).

Actinomycosis is a chronic suppurative infection caused by the Gram-positive bacillus *Actinomyces*, the species *Actinomyces israelii*, which is a member of the endogenous flora often found in the teeth, oropharynx, gastrointestinal tract, and female genital tract, being the most common agent in humans^(1)^.

The most commonly affected area is the cervicofacial region (in 50-65% of cases), followed by the thorax (in 15-30%) and the abdomen/pelvis (in 20%). However, within the cervicofacial region, the maxilla is the least commonly affected site, accounting for only 0.5-9.0% of cases in the head and neck. Bone involvement is even more rare, osteomyelitis being sporadic or secondary to infection at primary sites^(2-4)^. Risk factors for cervicofacial involvement include inadequate oral hygiene, trauma to the oral mucosa, chronic tonsillitis, otitis, mastoiditis, and osteonecrosis induced by radiotherapy or bisphosphonates. It is of note that, different than what is observed for the other affected sites, cervicofacial infection with *Actinomyces* sp. occurs more commonly in patients who are immunocompetent^(2,3)^.

In its acute form, actinomycosis usually manifests as edema of the soft tissues, together with the formation of masses and abscesses, evolving, chronically, to dissemination of the infection to the adjacent soft tissues, then the fascial planes, externalizing itself through fistulas of the skin and paranasal sinuses. However, it is rarely seen in combination with osteomyelitis^(3)^.

On computed tomography, actinomycosis appears as a mass with ill-defined borders, soft-tissue density, and contrast enhancement, together with fluid collections and fistulas. The differential diagnosis includes fungal ulcers, carcinoma, idiopathic midline granuloma, and osteomyelitis of the maxilla caused by other germs^(5)^. In the histopathological analysis, hematoxylin-eosin staining reveals chronic abscess with polymorphonuclear leukocytes, granulation tissue and fibrosis, Grocott’s staining revealing colonies of bacilli forming “sulfur granules”, which represent tangled filaments of *Actinomyces*, present in abscesses, exudates of the sinus tract, or tissues infiltrated by the lesions^(3,6)^.

Penicillin G is the drug of choice for the treatment of actinomycosis, requiring long courses of antibiotic therapy. Surgical management is reserved for the drainage of bulky abscesses, marsupialization of chronically infected sinus tracts, excision of fibrotic lesions, and debridement of necrotic bone tissue^(2)^.

Therefore, despite its rarity, it is important to bear actinomycosis of the maxilla in mind as a differential diagnosis, mainly in cases of aggressive lesions of the mouth related to the abovementioned predisposing factors.
